# Supplementary material for: Compact zinc finger architecture utilizing toxin-derived cytidine deaminases for highly efficient base editing in human cells
Source: Nat Commun. 2024 Feb 15;15:1181. doi: 10.1038/s41467-024-45100-w (PMC10869815; doi:10.1038/s41467-024-45100-w)
Supplement: Supplementary file 1 — Supplementary Information [file 41467_2024_45100_MOESM1_ESM.pdf]

Compact zinc finger architecture utilizing toxin-derived cytidine deaminases for highly efficient base editing in human cells

Friedrich Fauser[1], Bhakti N Kadam[1], Sebastian Arangundy-Franklin[1], Jessica E Davis[1], Vishvesha Vaidya[1], Nicola J Schmidt[1], Garrett Lew[1], Danny F Xia[1], Rakshaa Mureli[1], Colman Ng[1], Yuanyue Zhou[1], Nicholas A Scarlott[1], Jason Eshleman[1], Yuri R Bendaña[1], David A Shivak[1], Andreas Reik[1], Patrick Li[1], Gregory D Davis[1] and Jeffrey C Miller[1,2]

[1]Sangamo Therapeutics, Inc., Brisbane, CA, USA

[2]Contact: jmiller@sangamo.com

LIST OF SUPPLEMENTARY FIGURES

Figure S1 | Developing ZF-CBEs for CCR5.
Figure S2 | Developing ZF-CBEs for CIITA.
Figure S3 | Identification and characterization of alternative dsDNA deaminase domains for ZF-CBEs.
Figure S4 | TDD14-derived ZF-CBEs targeted to three additional sites within the CIITA locus
Figure S5 | Effect of different ZF-TDD linkers on base editing properties.
Figure S6 | Activity comparison of selected ZF-CBEs across the entire base editing window.
Figure S7 | Activity comparison of ZF-CBE and ZF-CBE-nickases in human T cells.
Figure S8 | Base editing in T cells at off-target sites using rhAmpSeq™.
Figure S9 | Off-target analysis of ZF-free CBEs in K562 cells.
Figure S10 | Flow cytometry gating strategy.
Figure S11 | SDS-PAGE gel of purified ZF-CBEs.
Figure S12 | Targeting ZF-CBE-nickases to EMILIN2.
Figure S13 | Targeting ZF-CBE-nickases to TRAM1L1.
Figure S14 | Targeting ZF-CBE-nickases to COL5A1.
Figure S15 | Targeting ZF-CBE-nickases to HBB.
Figure S16 | EMILIN2, TRAM1L1, COL5A1, and HBB ZF-CBE performance in T cells.

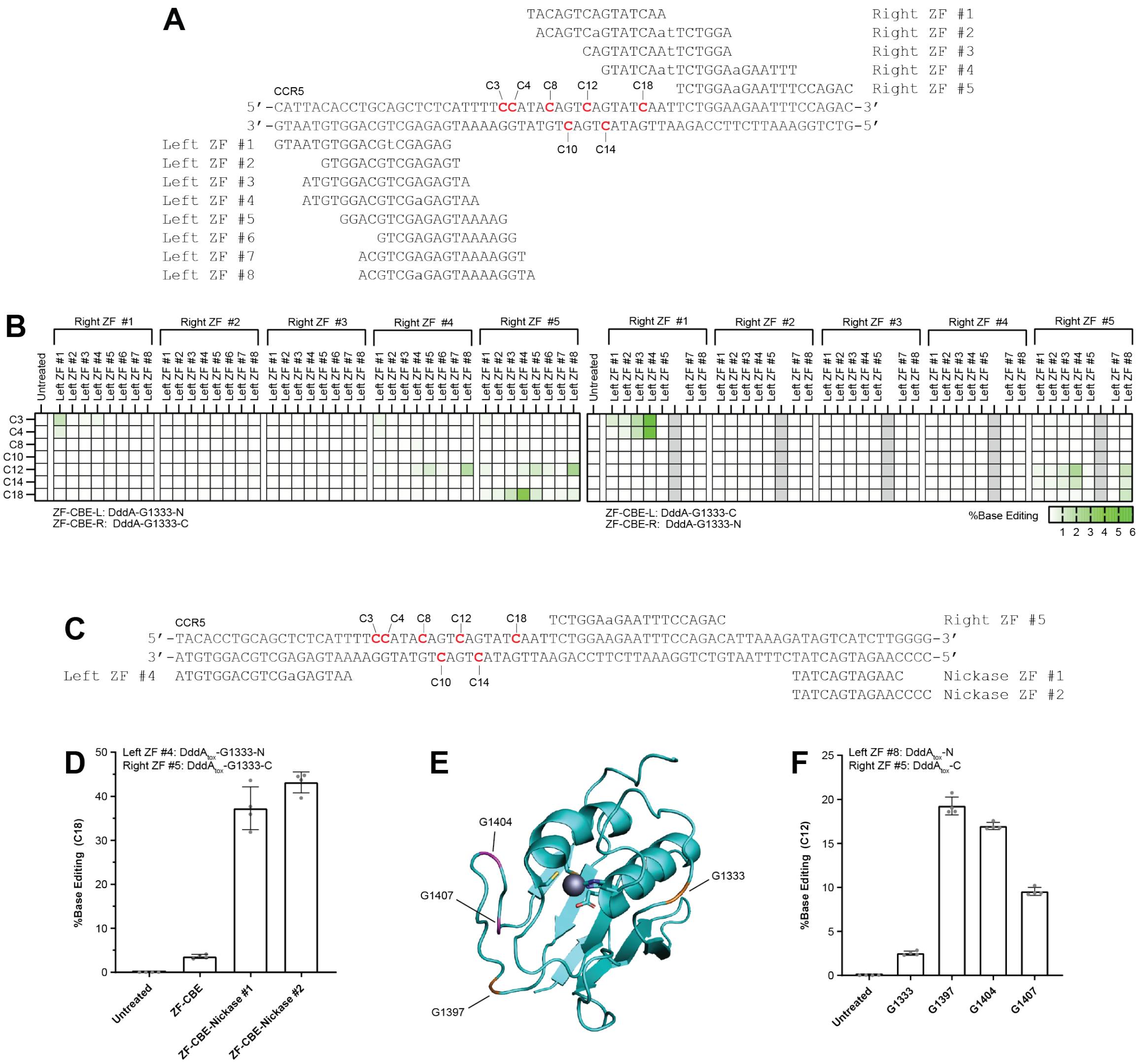

Supplementary Figure 1. Developing ZF-CBEs for CCR5. a) Grid of eight left and five right ZF arrays that were fused to split fragments of DddA$_{tox}$-G1333 to generate functional ZF-CBEs. Both split orientations were tested for all possible left-right pairings and the full dataset is summarized in panel **b**. Note that Left ZF #4 and Right ZF #5 were selected for the studies presented in **Figure 1** and panel **c**. Left ZF #8 and Right ZF #5 were selected for the studies presented in panel **f. b)** Activity comparison of all tested ZF-CBE pairs. Data are presented as the mean from two biological replicates (five replicates for untreated sample). For full dataset and plotted data values, see **supplementary data 1**. **c)** Two different ZF nickase constructs that can each pair with Right ZF #5 were generated. **d)** Activity comparison of the ZF-CBE constructs shown in **Figure 1a** with and without nicking using Nickase ZF #1 and Nickase ZF #2 from panel **c**. Data are presented as the mean ± s.d. from four biological replicates. For plotted data values, see **supplementary data 2**. Note that the Nickase ZF #2 data was selected for the studies presented in **Figure 1**. **e)** Structural model of DddA$_{tox}$. DddA$_{tox}$ was split at the peptide bond between G1333, G1397, G1404 and G1407 and the following residue. **f)** Activity comparison for different DddA$_{tox}$ split variants. Data are presented as the mean ± s.d. from four biological replicates. For plotted data values, see **supplementary data 3**. Source data are provided as a Source Data file.

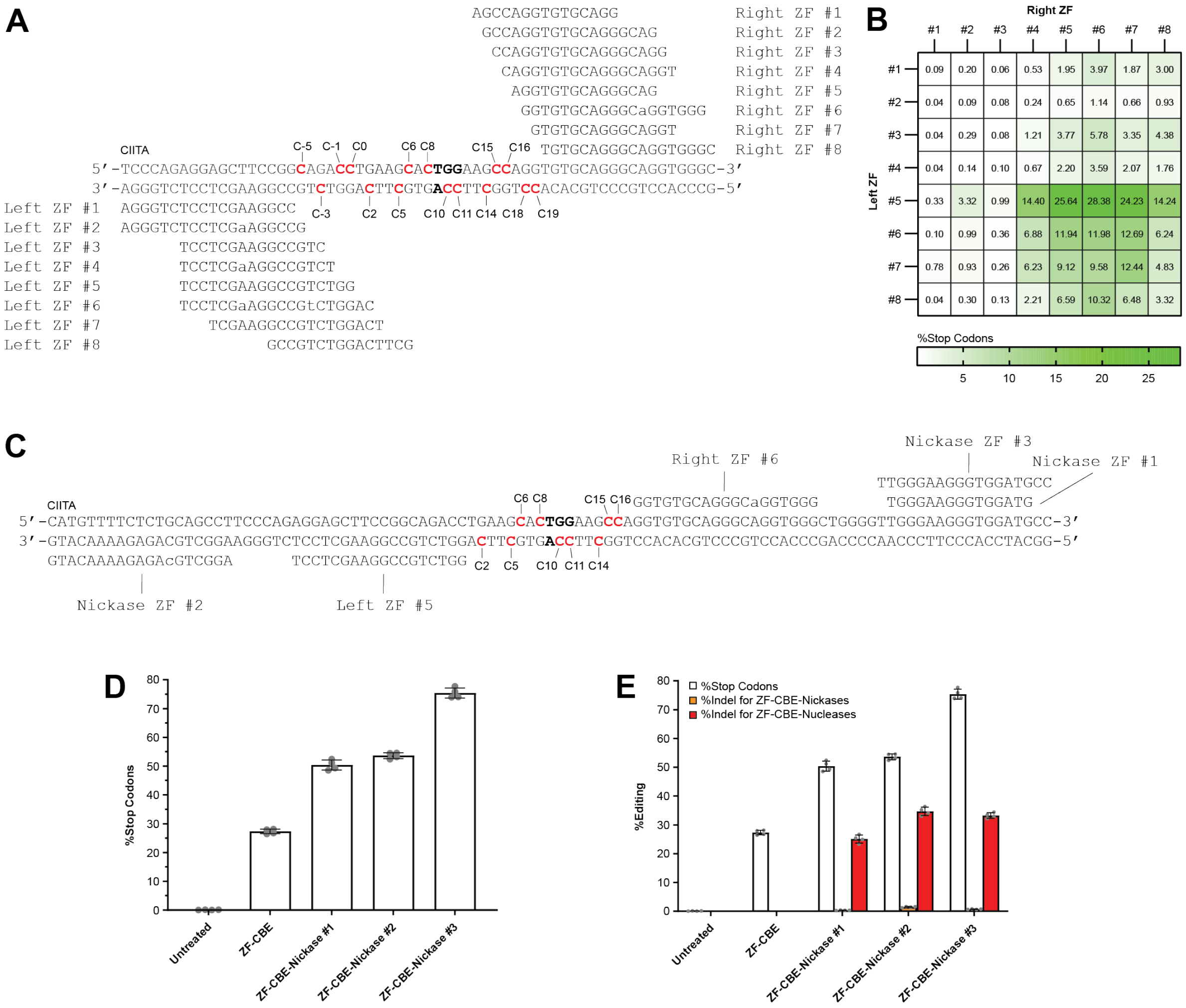

Supplementary Figure 2. Developing ZF-CBEs for CIITA. a) Grid of eight left and eight right ZF arrays that were fused to split fragments of $DddA_{tox}$-G1404 to generate functional ZF-CBEs. The C-terminal fragment of $DddA_{tox}$-G1404 was fused to the left ZF arrays, the N-terminal fragment of $DddA_{tox}$-G1404 was fused to the right ZF arrays, all possible left-right pairings were tested, and the full dataset is summarized in panel **b**. Note that Left ZF #5 and Right ZF #6 were selected for the studies presented in **Figure 2** and panel **c**, and that cytosine numbering in panel **a** is based on the base editing window of this pair. **b)** Activity comparison of the ZF-CBE constructs shown in panel **a**. Data are presented as the mean from two biological replicates (16 replicates for untreated sample). For plotted data values, see **supplementary data 4**. **c)** Two ZF arrays were designed to work together with Right ZF #6 as a ZF nickase as depicted in **Figure 2**. Similarly, one ZF array was designed to work together with Left ZF #5 as a ZF nickase. **d)** Activity comparison of the ZF-CBE constructs shown in **Figure 2a** with and without nicking using Nickase ZF #1, Nickase ZF #2 and Nickase ZF #3 from panel **c**. Note that the Nickase ZF #3 data was selected for the studies presented in **Figure 2**. Data are presented as the mean ± s.d. from four biological replicates. For plotted data values, see **supplementary data 5**. **e)** Indel activity comparison of ZF-CBE, ZF-CBE-nickases and ZF-CBE-Nucleases. Data are presented as the mean ± s.d. from four biological replicates. For plotted data values, see **supplementary data 5**. Source data are provided as a Source Data file.

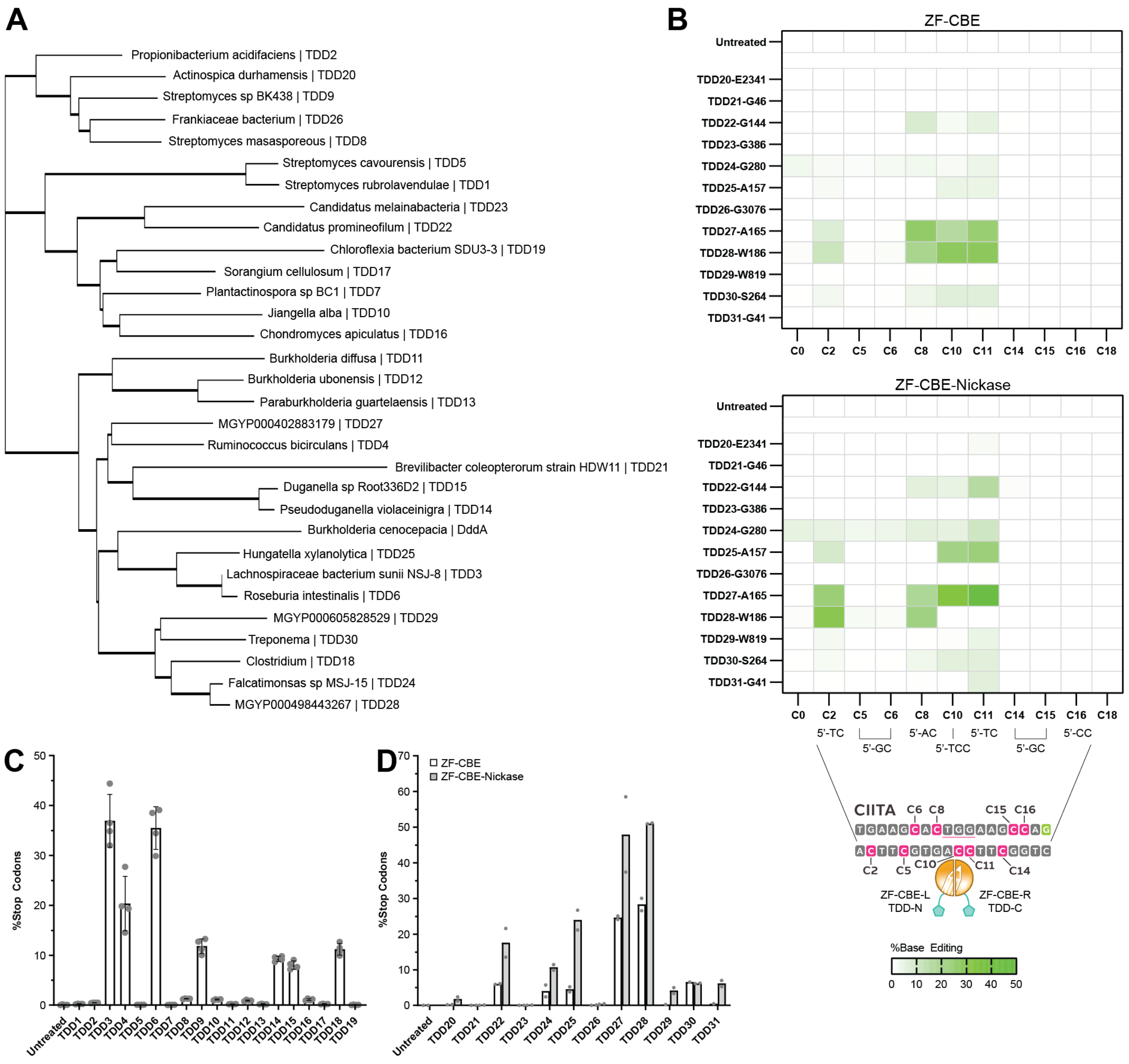

Supplementary Figure 3. Identification and characterization of alternative dsDNA deaminase domains for ZF-CBEs. a) Phylogenetic tree representing the evolutionary relationship between $DddA_{tox}$ and TDD1 to TDD30. **b)** Activity comparison of TDD20 to TDD31. Note that TDD31 is a consensus sequence and not a naturally derived sequence. Up to three different split variants of TDD20 to TDD31 were tested as ZF-CBEs and ZF-CBE-nickases, and the most active variants are shown in panel **b**. Data are presented as the mean from two biological replicates for TDD20 to TDD31, except for the no nickase controls of TDD29, TDD30 and TDD31 which have only been tested once. For the full dataset and plotted data values, see **supplementary data 7**). **c)** Activity comparison of the most active (%stop codons) TDD1 to TDD19-derived ZF-CBEs from **Figure 3b**. TDD1: Left-G2278-C, Right-G2278-N; TDD2: Left-G1794-N, Right-G1794-C; TDD3: Left-N94-N, Right-N94-C; TDD4: Left-A229-C, Right-A229-N; TDD5: Left-G2299-N, Right-G2299-C; TDD6: Left-R2385-N, Right-R2385-C; TDD7: Left-G102-N, Right-G102-C; TDD8: Left-T2175-N, Right-T2175-C; TDD9: Left-2179-N, Right-2179-C; TDD10: Left-G1746-C, Right-G1746-

N; TDD11: Left-G1430-C, Right-G1430-N; TDD12: Left-A1488-C, Right-A1488-N; TDD13: Left-A1477-C, Right-A1477-N; TDD14: Left-G43-C, Right-G43-N; TDD15: Left-G315-C, Right-G315-N; TDD16: Left-G1264-C, Right-G1264-N; TDD17: Left-G2156-C, Right-G2156-N; TDD18: Left-A250-C, Right-A250-N and TDD19: Left-G234-N, Right-G234-C. Data are presented as the mean ± s.d. from four biological replicates. For the full dataset and plotted data values, see **supplementary data 6**. **d)** Activity comparison of TDD20 to TDD31 with and without nicking. Data are presented as the mean from two biological replicates. For the full dataset and plotted data values, see **supplementary data 7**. Source data are provided as a Source Data file.

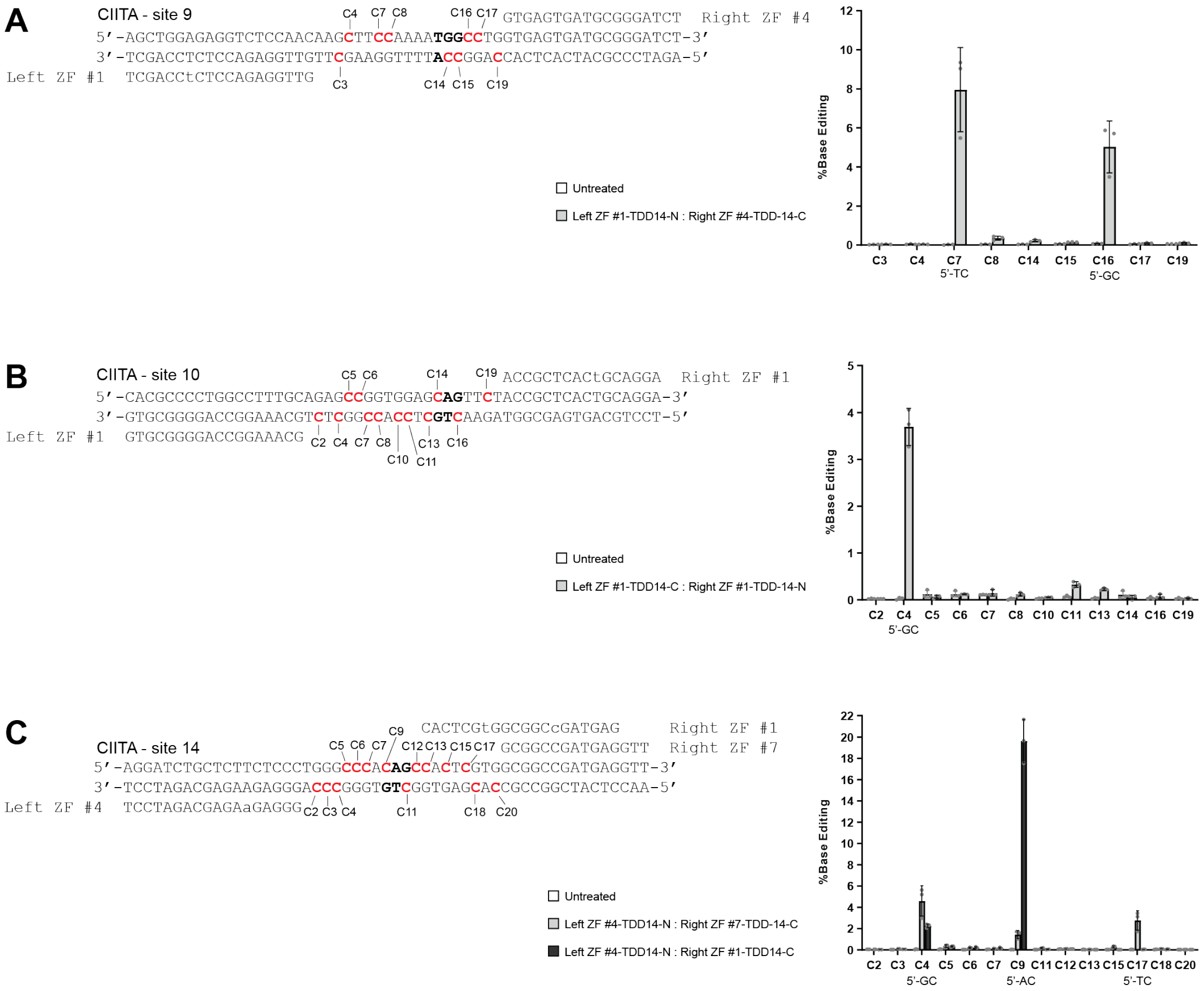

Supplementary Figure 4. TDD14-derived ZF-CBEs targeted to three additional sites within the CIITA locus. **a)** Activity comparison of one ZF-CBE pair targeted to CIITA site 9. Data are presented as the mean ± s.d. from three biological replicates. **b)** Activity comparison of one ZF-CBE pair targeted to CIITA site 10. Data are presented as the mean ± s.d. from three biological replicates. **c)** Activity comparison of two ZF-CBE pairs targeted to CIITA site 14. Data are presented as the mean ± s.d. from three biological replicates. Note that the TDD14-G43 split variant was used for data shown in panels **a-c**. For the full datasets and plotted data values, see **supplementary data 8**. Source data are provided as a Source Data file.

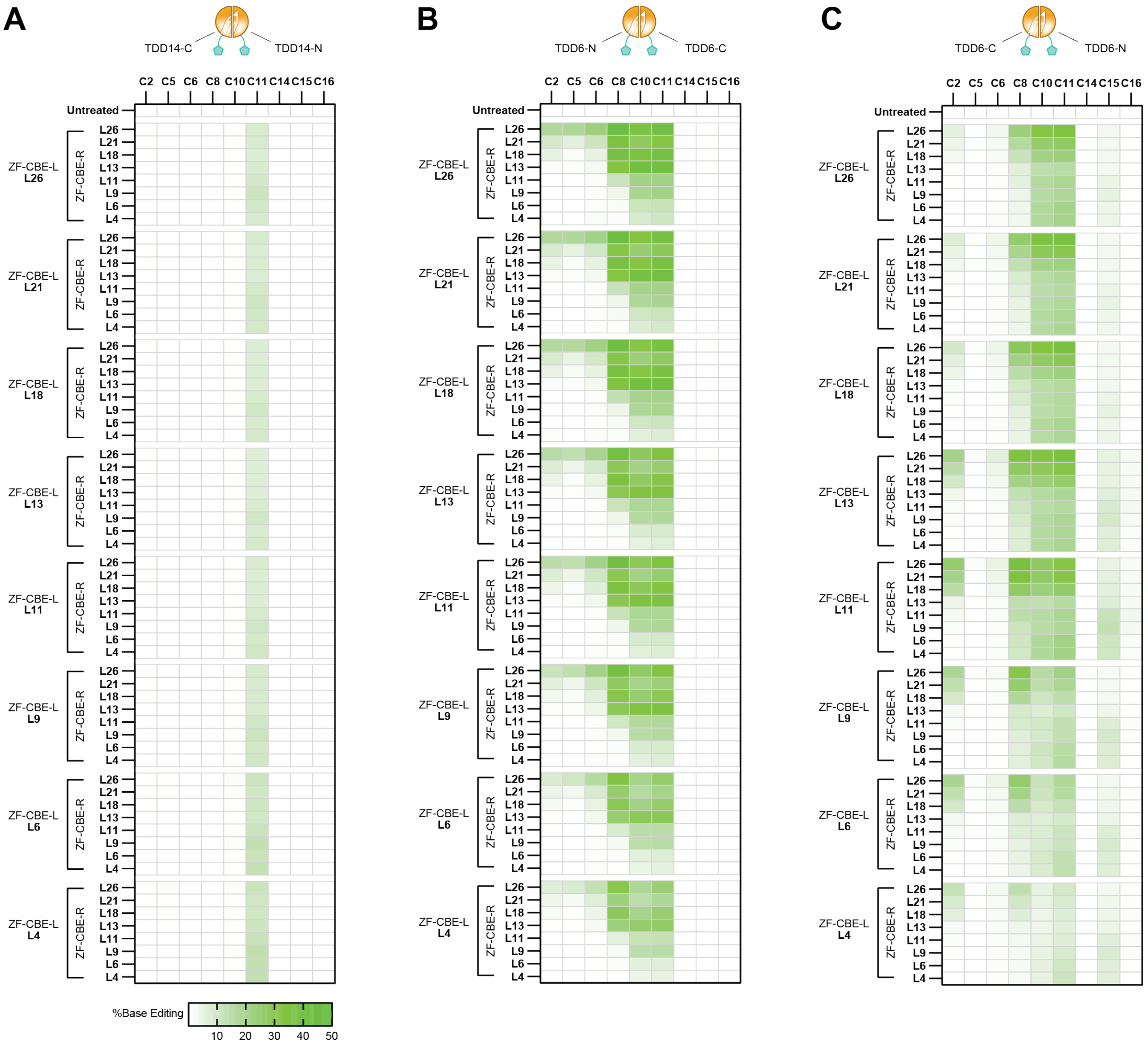

Supplementary Figure 5. Effect of different ZF-TDD linkers on base editing properties. Eight different linkers were tested in the context of previously established TDD14 and TTD6 ZF-CBEs targeting the CIITA locus (**see Figure 3** and **supplementary figure 2**). L26 describes a 26aa linker (LRGSQLVKSKSEAAARGGGGSGGGGS) used in the parental constructs. L21 (LRGSQLVKSKSEAAARGGGGS), L18 (LRGSQLVKSKSEAAARGS), L13 (LRGSQLVKSKSGS), L11 (LRGSQLVKSGS), L9 (LRGSQLVGS, L6 (LRGSGS) and L4 (LRGS are shorter variants of L26. **a)** TDD14 linker study. The C-terminal fragment of TDD14 was fused to ZF-CBE-L and the N-terminal fragment of TDD14 was fused to ZF-CBE-R. **b-c)** TDD6 linker study. In panel **b**, the N-terminal fragment of TDD6 was fused to ZF-CBE-L and the C-terminal fragment of TDD6 was fused to ZF-CBE-R, while the opposite orientation was tested in panel **c**. Data in panels **a-c** are presented as the mean from four biological replicates. For the full dataset and plotted data values, see **supplementary data 9**. Source data are provided as a Source Data file.

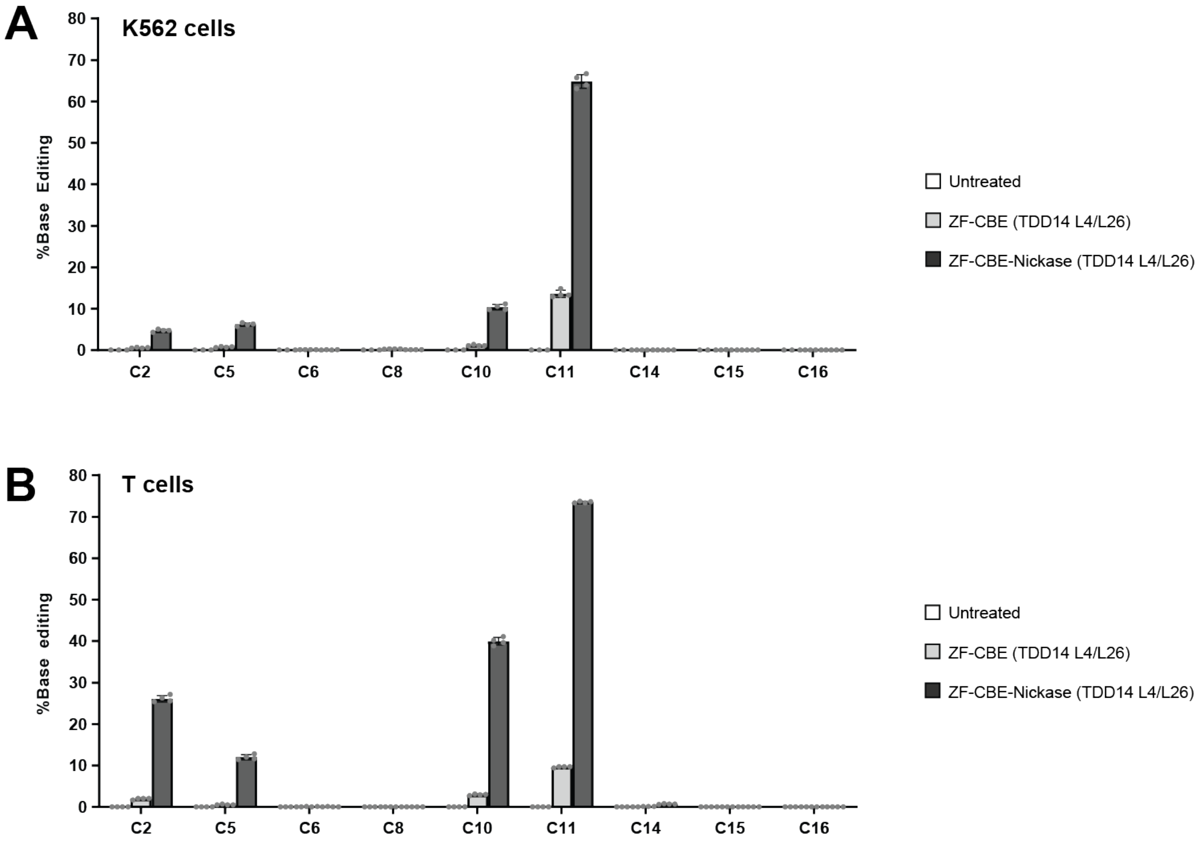

Supplementary Figure 6. Activity comparison of selected ZF-CBEs across the entire base editing window. a) Activity comparison of ZF-CBEs shown in **Figure 3c** in K562 cells. Data are presented as the mean ± s.d. from four biological replicates (three for control sample). For the full dataset and plotted data values, see **supplementary data 10**. **b)** Activity comparison of ZF-CBEs shown in **Figure 4c** in T cells. Data are presented as the mean ± s.d. from four biological replicates. For the full dataset and plotted data values, see **supplementary data 12**. Source data are provided as a Source Data file.

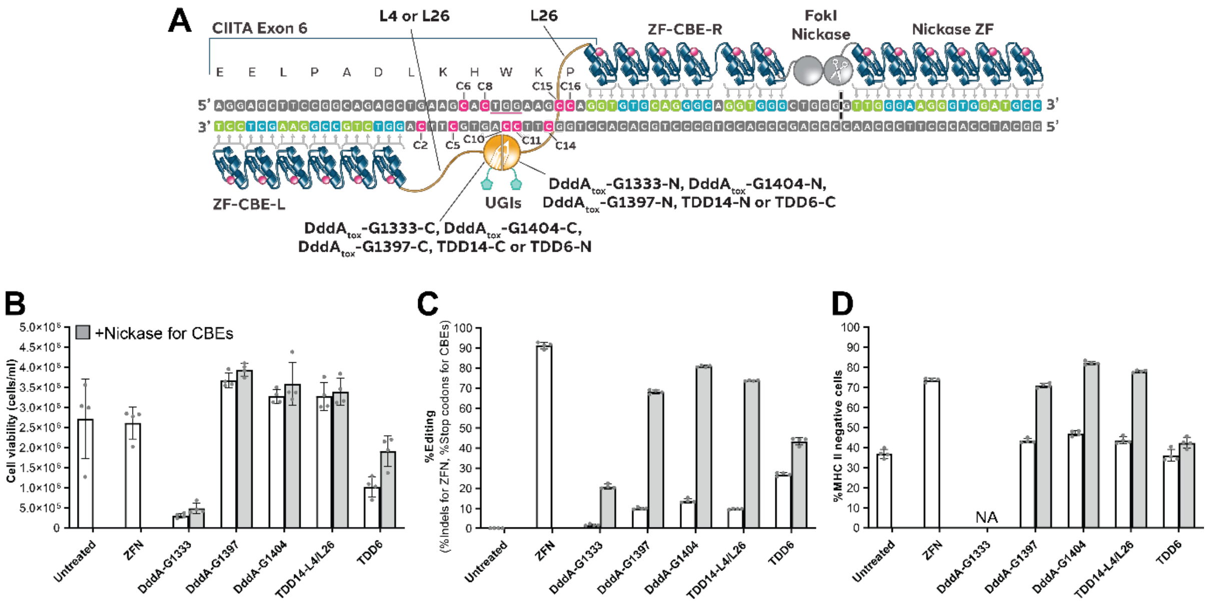

Supplementary Figure 7. Activity comparison of ZF-CBE and ZF-CBE-nickases in human T cells. a) Sketch of a ZF-CBE-nickase bound to its target site within the human CIITA locus as described in **Figure 2a**. The left ZF-CBE arm is fused to the C-terminal fragment of DddA$_{tox}$-G1333, or the C-terminal fragment of DddA$_{tox}$-G1397, or the C-terminal fragment of DddA$_{tox}$-G1404 (see also **Figure 2**), or the C-terminal fragment of TDD14 (see **Figure 3**), or the N-terminal fragment of TDD6 (see **Figure 3**), while the right ZF-CBE arm is fused to their matching fragments. Note that linker L26 was used if not mentioned otherwise. **b-d)** comparison of different ZF-CBEs and ZF-CBE-nickases to a ZFN control. Measurements were taken 10 days post transfection. **b)** Cell viability assay measuring cell density. **c)** PCR-based NGS assay measuring %Stop codons for ZF-CBE-nickases or %Indels for the ZFN control construct. **d)** FACS-based assay to measure MHC II reduction. Data are presented as the mean ± s.d. from four biological replicates. For plotted data values in panels **b-d**, see **supplementary data 11-13**. Source data are provided as a Source Data file.

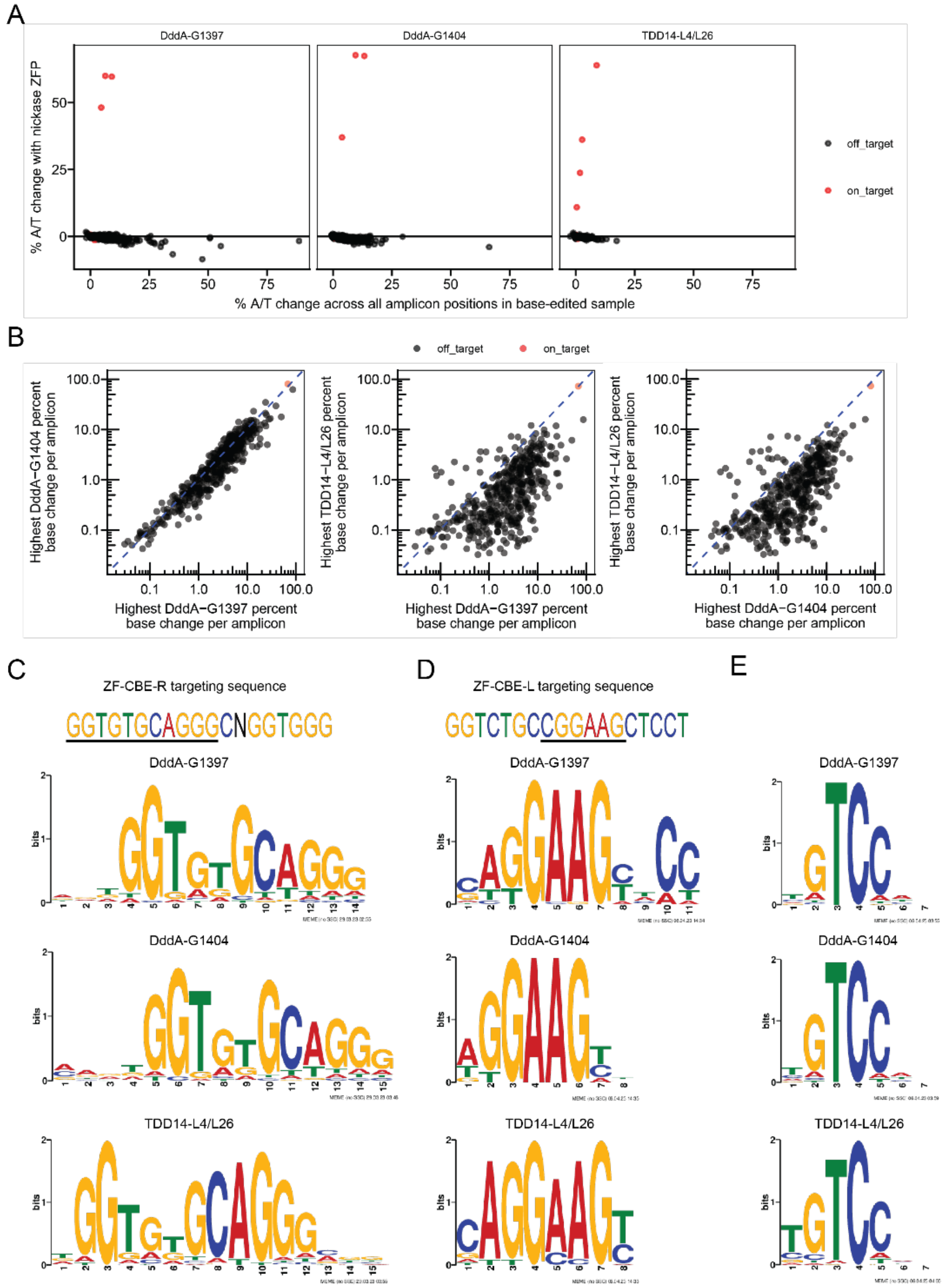

Supplementary Figure 8. Base editing in T cells at off-target sites using rhAmpSeq™. a) The effect of the nickase on base editing signal across all positions of all rhAmpSeq™ amplicons. The x axis indicates cytosine base editing without the nickase as indicated by % change in A or T nucleotides across each amplicon when compared to unedited samples. The y axis is the % change in cytosine base editing signal when the nickase arm was co-transfected. Edits at CIITA are indicated in red with all other targets indicated in black. **b)** Comparison of base editing measurements of each rhAmpSeq™ amplicon between ZF-CBEs with nickase. The CIITA on-target amplicon is indicated in red and all other amplicons are indicated in black. x=y is indicated as a dashed blue line. **c)** The top motif identified in off-target sites from the rhAmpSeq™ that indicated ≥ 1% base editing indicated strong similarity to the ZF-CBE-R CIITA binding site. Meme from memesuite was used to identify the motifs (DddA-G1397 E-value: 1.3e-345, DddA-G1404

E-value: 3.7e-340, TDD14 E-value: 1.3e-212). **d)** A motif with similarity to a portion of the ZF-CBE-L CIITA binding site was also identified when restricting motif search to positions +3 to +25 and –3 to –25 from the position with the highest base editing per amplicon within amplicons with ≥ 1% base editing. Meme from memesuite was used to identify the motifs (DddA-G1397 E-value: 1.5e-022, DddA-G1404 E-value: 3.8e-016, TDD14 E-value: 2.7e-010). **e)** A motif search centered on the position with the highest base editing per amplicon within amplicons with ≥ 1% base editing identified the cytosine base editing context preference of each ZF-CBE. Meme from memesuite was used to identify the motifs (DddA-G1397 E-value: 3.0e-543, DddA-G1404 E-value: 2.1e-517, TDD14 E-value: 2.8e-235). Source data are provided as a Source Data file.

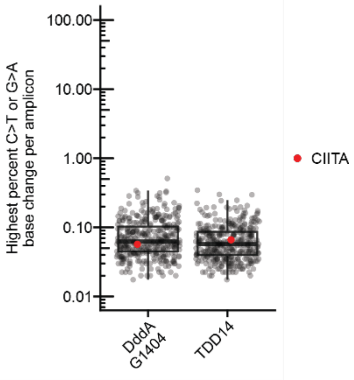

Supplementary Figure 9. Off-target analysis of ZF-free CBEs in K562 cells. Base editing at off-target sites identified in the genome-wide specificity assay was measured in K562 using the same rhAmpSeq™ panel as in **Figure 5c**. Both DddAtox-G1404 and TDD14 without ZFPs (but still retaining 1 UGI) were screened for base editing at 385 candidate off-target sites identified in all genome-wide specificity assays. The highest percent change of a C>T or G>A conversion from the control sample per each amplicon is shown as an indicator of cytosine base editing on either strand. Box plot elements: Center line, median; box limits, upper and lower quartiles; whiskers, 1.5x interquartile range; all data points shown. Source data are provided as a Source Data file.

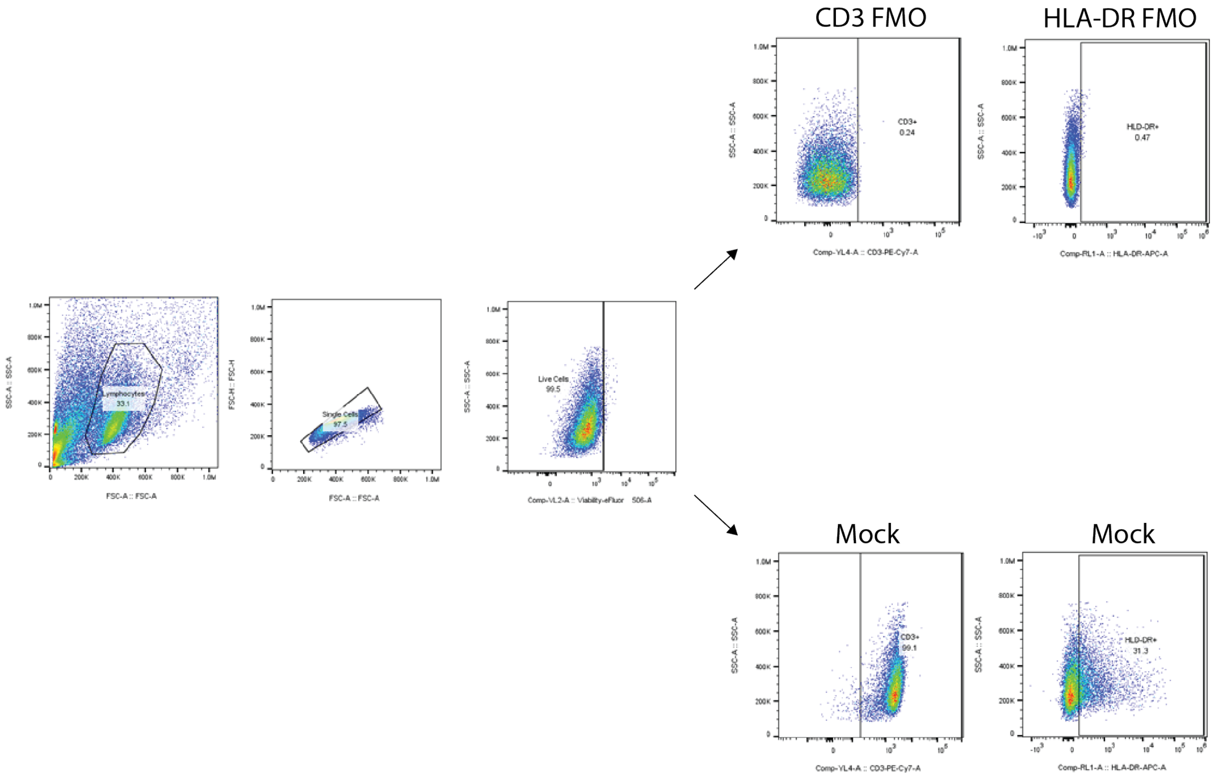

Supplementary Figure 10. Flow cytometry gating strategy. Gating strategy for the flow cytometry experiment with T cells is shown in the dot plots. Please note that 2.5ul of Isotype e506 (Thermo, 69-4321-82) was used instead of e506 as a viability stain for compensation beads.

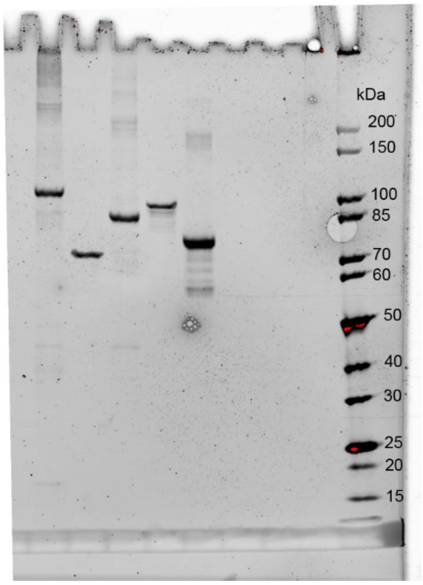

Supplementary Figure 11. SDS-PAGE gel of purified ZF-CBEs. Purified ZF-CBEs used in the genome-wide specificity assays were confirmed for their size and purity using an SDS-PAGE gel. From left to right: ZF-CBE-R-ELD-D450N (106 kDa), ZF-CBE-L (72 kDa), Nickase ZF-KKR (90 kDa), TDD14-derived ZF-CBE-R-ELD-D450N (98 kDa), TDD14-derived ZF-CBE-L (77 kDa).

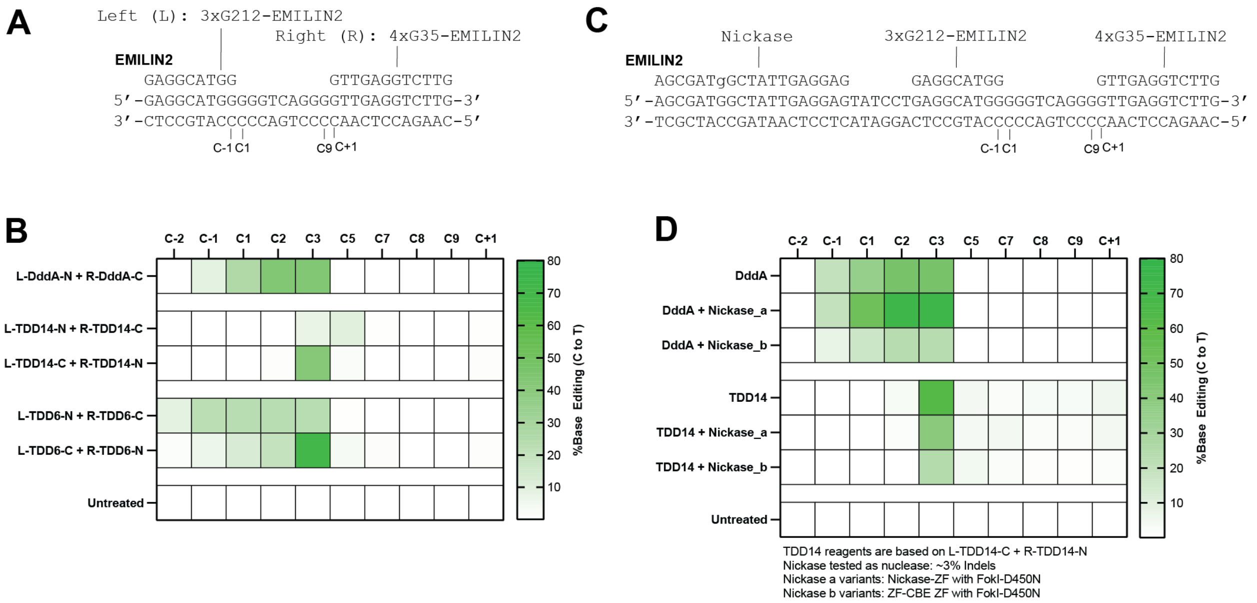

Supplementary Figure 12. Targeting ZF-CBE-nickases to EMILIN2. To demonstrate generalizability, we designed TDD6 and TDD14-derived ZF-CBEs and ZF-CBE-nickases for EMILIN2, a target site previously described by Willis *et al.* (2022). **a)** Nuclear EMILIN2 target site in human K562 cells. 3xG212-EMILIN2 and 4xG35-EMILIN2 depict binding sites of ZFPs established by Willis *et al.* (2022). **b)** Activity comparison of the parental DddA-derived (DddA: T1381I, E1396K, T1413I) base editing construct (Willis *et al.*, 2022), and TDD variants thereof. Data are presented as the mean from two biological replicates. For the full dataset and plotted data values, see **supplementary data 27**. **c)** A nickase ZFP was designed using publicly available design information to work together with the established ZF-CBE ZF pair (**supplementary data 33**). **d)** Activity comparison of selected ZF-CBE-nickases. Data are presented as the mean from four biological replicates. For the full dataset and plotted data values, see **supplementary data 27.** All experiments were performed in human K562 cells. Source data are provided as a Source Data file.

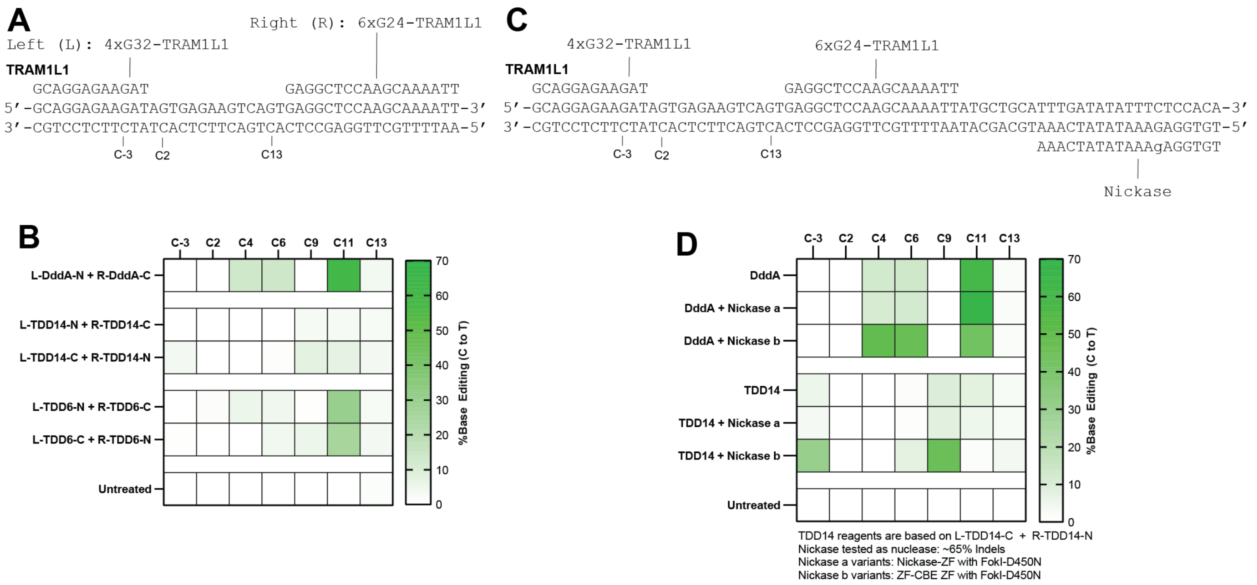

Supplementary Figure 13. Targeting ZF-CBE-nickases to TRAM1L1. To demonstrate generalizability, we designed TDD6 and TDD14-derived ZF-CBEs and ZF-CBE-nickases for TRAM1L1, a target site previously described by Willis *et al.* (2022). **a)** Nuclear TRAM1L1 target site in human K562 cells. 4xG32-TRAM1L1 and 6xG24-TRAM1L1 depict binding sites of ZFPs established by Willis *et al.* (2022). **b)** Activity comparison of the parental DddA-derived (DddA: T1381I, E1396K, T1413I) base editing construct (Willis *et al.*, 2022), and TDD variants thereof. Data are presented as the mean from two biological replicates. For the full dataset and plotted data values, see **supplementary data 28**. **c)** A nickase ZFP was designed using publicly available design information to work together with the established ZF-CBE ZF pair (**supplementary data 33**). **d)** Activity comparison of selected ZF-CBE-nickases. Data are presented as the mean from four biological replicates. For the full dataset and plotted data values, see **supplementary data 28.** All experiments were performed in human K562 cells. TRAM1L1 data was normalized to a control to remove PCR background (**supplementary figure 28**). Source data are provided as a Source Data file.

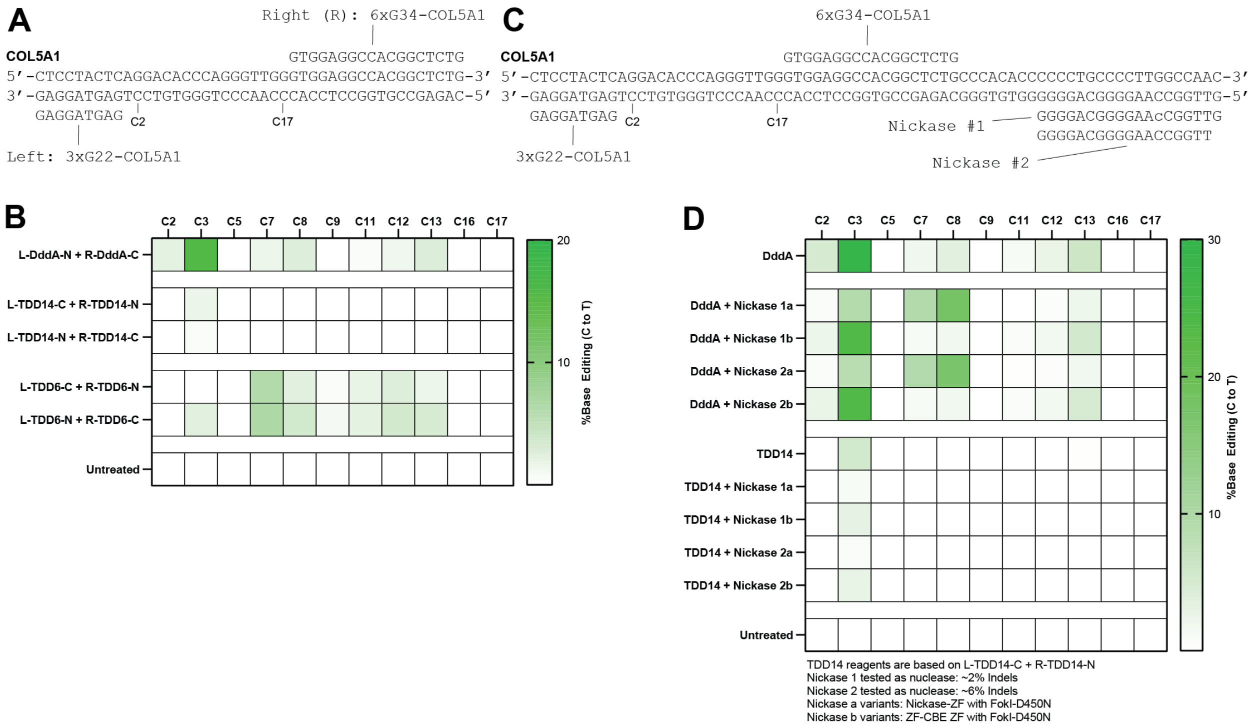

Supplementary Figure 14. Targeting ZF-CBE-nickases to COL5A1. To demonstrate generalizability, we designed TDD6 and TDD14-derived ZF-CBEs and ZF-CBE-nickases for COL5A1, a target site previously described by Willis *et al.* (2022). **a)** Nuclear COL5A target site in human K562 cells. 3xG22-COL5A1 and 6xG34-COL5A1 depict binding sites of ZFPs established by Willis *et al.* (2022). **b)** Activity comparison of the parental DddA-derived (DddA: T1381I, E1396K, T1413I) base editing construct (Willis *et al.*, 2022), and TDD variants thereof. Data are presented as the mean from two biological replicates. For the full dataset and plotted data values, see **supplementary data 29**. **c)** Two nickase ZFPs were designed using publicly available design information to work together with the established ZF-CBE ZF pair (**supplementary data 3**). **d)** Activity comparison of selected ZF-CBE-nickases. Data are presented as the mean from four biological replicates. For the full dataset and plotted data values, see **supplementary data 29.** All experiments were performed in human K562 cells. Source data are provided as a Source Data file.

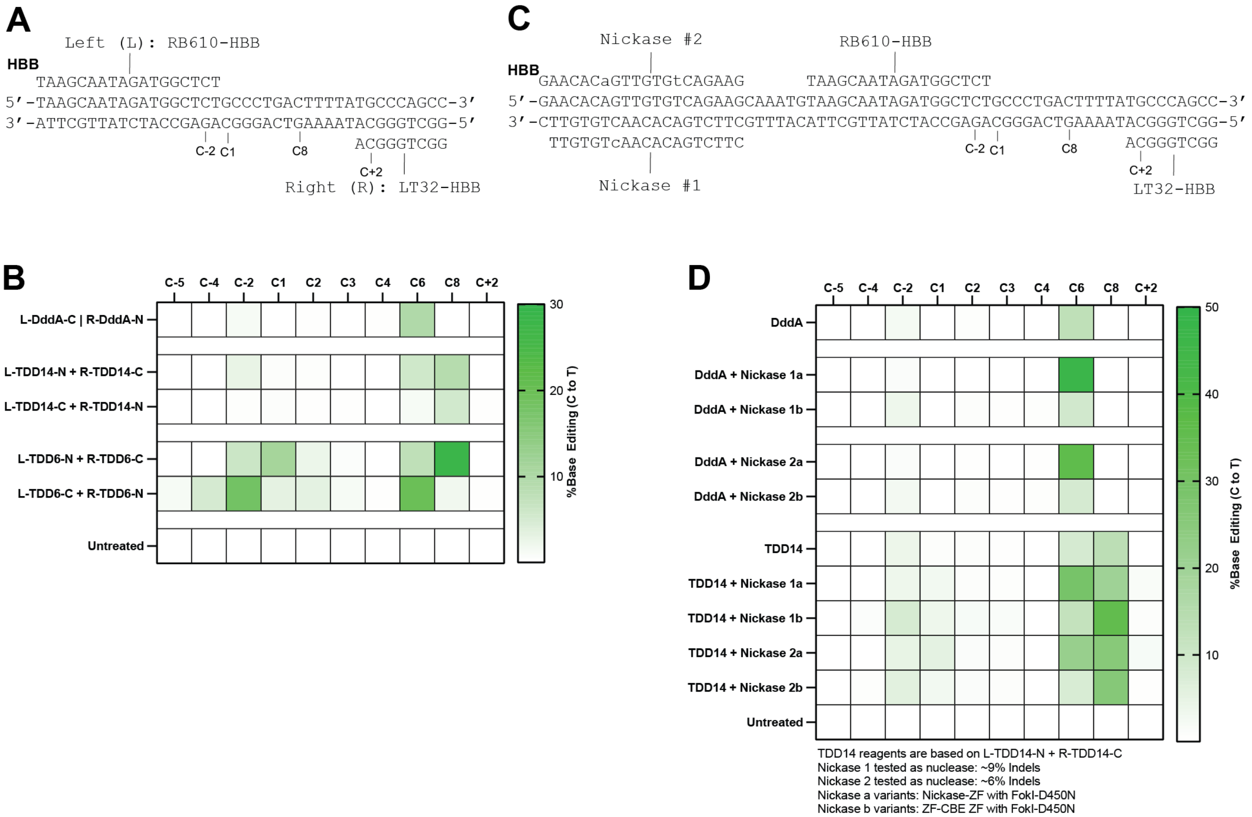

Supplementary Figure 15. Targeting ZF-CBE-nickases to HBB. To demonstrate generalizability, we designed TDD6 and TDD14-derived ZF-CBEs and ZF-CBE-nickases for HBB, a target site previously described by Willis *et al.* (2022). **a)** Nuclear COL5A target site in human K562 cells. RB610-HBB and LT32-HBB depict binding sites of ZFPs established by Willis *et al.* (2022). **b)** Activity comparison of the parental DddA-derived (DddA: T1381I, E1396K, T1413I) base editing construct (Willis *et al.*, 2022), and TDD variants thereof. Data are presented as the mean from two biological replicates. For the full dataset and plotted data values, see **supplementary data 30**. **c)** Two nickase ZFPs were designed using publicly available design information to work together with the established ZF-CBE ZF pair (**supplementary data 33**). **d)** Activity comparison of selected ZF-CBE-nickases. Data are presented as the mean from four biological replicates. For the full dataset and plotted data values, see **supplementary data 30.** All experiments were performed in human K562 cells. Source data are provided as a Source Data file.

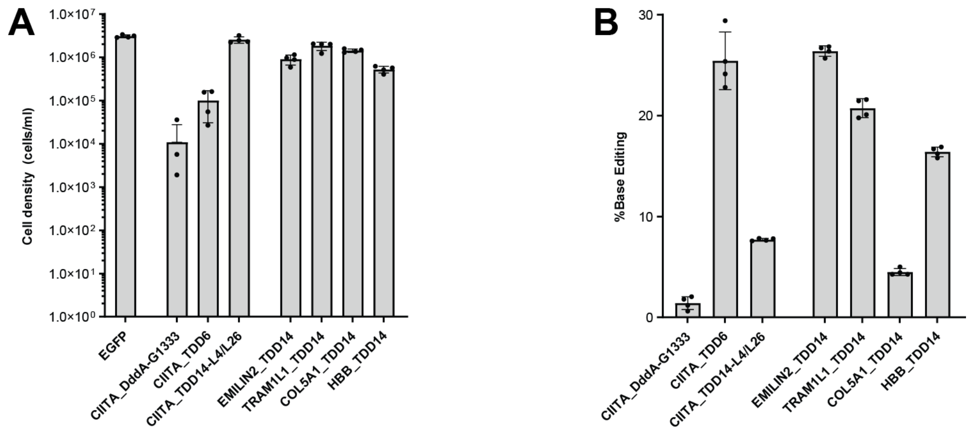

Supplementary Figure 16. EMILIN2, TRAM1L1, COL5A1, and HBB ZF-CBE performance in T cells. We tested TDD-14-derived ZF-CBEs targeted to four different nuclear target sites (see **supplementary figures 12-15**) in T cells to investigate whether they cause any substantial effect on cell viability. We used the same TDD14 split orientation as in **supplementary figures 13-16c. a)** Cell viability assay measuring cell density. We tested the shown TDD14 ZF-CBEs side-by-side with ZF-CBE reagents that previously impacted cell growth in T cells (see **figure 4** for comparison). **b)** PCR-based NGS assay measuring on-target base editing activity. Graph shows the maximum editing efficiency in each base editing window (EMILIN2: C3, TRAM1L1: C9, COL5A1: C3, HBB: C8, CIITA: % stop codons). All measurements were taken 10 days post transfection. Data are presented as the mean ± s.d. from four biological replicates. For plotted data values, see **supplementary data 31 and 32**. Source data are provided as a Source Data file.
