# Supplementary material for: Compact zinc finger architecture utilizing toxin-derived cytidine deaminases for highly efficient base editing in human cells
Source: Nat Commun. 2024 Feb 15;15:1181. doi: 10.1038/s41467-024-45100-w (PMC10869815; doi:10.1038/s41467-024-45100-w)
Supplement: Supplementary file 2 — Reporting Summary [file 41467_2024_45100_MOESM2_ESM.pdf]

nature portfolio

Corresponding author(s): Jeffrey C. Miller

Last updated by author(s): Dec 14, 2023

Reporting Summary

Nature Portfolio wishes to improve the reproducibility of the work that we publish. This form provides structure for consistency and transparency in reporting. For further information on Nature Portfolio policies, see our Editorial Policies and the Editorial Policy Checklist.

Statistics

For all statistical analyses, confirm that the following items are present in the figure legend, table legend, main text, or Methods section.

n/a	Confirmed	
☐	☒	The exact sample size (*n*) for each experimental group/condition, given as a discrete number and unit of measurement
☐	☒	A statement on whether measurements were taken from distinct samples or whether the same sample was measured repeatedly
☒	☐	The statistical test(s) used AND whether they are one- or two-sided<br>*Only common tests should be described solely by name; describe more complex techniques in the Methods section.*
☒	☐	A description of all covariates tested
☒	☐	A description of any assumptions or corrections, such as tests of normality and adjustment for multiple comparisons
☐	☒	A full description of the statistical parameters including central tendency (e.g. means) or other basic estimates (e.g. regression coefficient) AND variation (e.g. standard deviation) or associated estimates of uncertainty (e.g. confidence intervals)
☒	☐	For null hypothesis testing, the test statistic (e.g. *F*, *t*, *r*) with confidence intervals, effect sizes, degrees of freedom and *P* value noted<br>*Give P values as exact values whenever suitable.*
☒	☐	For Bayesian analysis, information on the choice of priors and Markov chain Monte Carlo settings
☒	☐	For hierarchical and complex designs, identification of the appropriate level for tests and full reporting of outcomes
☒	☐	Estimates of effect sizes (e.g. Cohen's *d*, Pearson's *r*), indicating how they were calculated

Our web collection on statistics for biologists contains articles on many of the points above.

Software and code

Policy information about availability of computer code

Data collection	Standard manufacturer's software on the relevant scientific instruments was used to collect all data.
Data analysis	Custom software, e.g. described in Miller et al. (2019; https://doi.org/10.1038/s41587-019-0186-z), was used for analysis of all Illumina sequencing data. Sequence analysis also utilized fastq_quality_filter from the FASTX toolkit, Illumina bcl2fastq software, SeqPrep, Bowtie2, and Biopython pairwise2 global aligner. Custom computer scripts used to automate more standard portions of the data analysis pipeline are available upon request. Flow cytometry data was analyzed with FlowJo (v10.4, FlowJo LLC). GraphPad Prism 9, Adobe Illustrator 2022, Microsoft Excel 365 and RStudio were used for generating figures and tables. Motifs were identified from the rhAmpSeq™ results using meme (version 5.5.0) from memesuite.

For manuscripts utilizing custom algorithms or software that are central to the research but not yet described in published literature, software must be made available to editors and reviewers. We strongly encourage code deposition in a community repository (e.g. GitHub). See the Nature Portfolio guidelines for submitting code & software for further information.

Data

Policy information about availability of data

All manuscripts must include a data availability statement. This statement should provide the following information, where applicable:

- Accession codes, unique identifiers, or web links for publicly available datasets
- A description of any restrictions on data availability
- For clinical datasets or third party data, please ensure that the statement adheres to our policy

NCBI accession numbers of deaminase used in this study are listed in supplementary table 16. Illumina sequencing data underlying all key experiments are currently deposited in the NCBI Sequence Read Archive under accession code PRJNA1052081 and will be available before publication.

Research involving human participants, their data, or biological material

Policy information about studies with human participants or human data. See also policy information about sex, gender (identity/presentation), and sexual orientation and race, ethnicity and racism.

Reporting on sex and gender	NA
Reporting on race, ethnicity, or other socially relevant groupings	NA
Population characteristics	NA
Recruitment	NA
Ethics oversight	NA

Note that full information on the approval of the study protocol must also be provided in the manuscript.

Field-specific reporting

Please select the one below that is the best fit for your research. If you are not sure, read the appropriate sections before making your selection.

[x] Life sciences [ ] Behavioural & social sciences [ ] Ecological, evolutionary & environmental sciences

For a reference copy of the document with all sections, see nature.com/documents/nr-reporting-summary-flat.pdf

Life sciences study design

All studies must disclose on these points even when the disclosure is negative.

Sample size	No power calculations were performed to determine sample sizes. Samples were processed in independent triplicates or quadruplicates and in most cases this was sufficient to observe clear differences between samples. We chose this sample size since it is the common procedure in the field, and we have found it to produce reliable results for platform development purposes.
Data exclusions	A small number of individual replicates were excluded from analysis due to failed PCR reactions. Some samples were also excluded and listed as "NA" for failing one or more quality metrics described, e.g. failed cloning.
Replication	All of the experiments in figures 3, 4, and 5 of the main text are better controlled and/or better executed versions of preliminary experiments. In all cases the results from the unreported preliminary experiments and the reported final experiments are consistent. All experiments were found to be reproducible between different data sets collected across a timescale of several months between various researchers.
Randomization	Randomization was not necessary for any of these experiments because all experiments involved aliquots of cells from a homogeneous cell culture.
Blinding	Samples were not blinded because there was no subjective component in the data processing or sample handling.

Reporting for specific materials, systems and methods

We require information from authors about some types of materials, experimental systems and methods used in many studies. Here, indicate whether each material, system or method listed is relevant to your study. If you are not sure if a list item applies to your research, read the appropriate section before selecting a response.

Materials & experimental systems

n/a	Involved in the study	
☐	☒	Antibodies
☐	☒	Eukaryotic cell lines
☒	☐	Palaeontology and archaeology
☒	☐	Animals and other organisms
☒	☐	Clinical data
☒	☐	Dual use research of concern
☒	☐	Plants

Methods

n/a	Involved in the study	
☒	☐	ChIP-seq
☐	☒	Flow cytometry
☒	☐	MRI-based neuroimaging

Antibodies

Antibodies used	PECy7 anti-human CD3 antibody (Biolegend, catalog number 300420, lot number B370636); APC anti-HLA-DR antibody (Biolegend, catalog number 361714, lot number B289409). See Material & Methods for detail.
Validation	Antibodies were validated by the manufacturer, used in accordance with the manufacturer's website, and the specific concentrations used in our study are provided in the Material & Methods section.

Eukaryotic cell lines

Policy information about cell lines and Sex and Gender in Research

Cell line source(s)	K562 cells obtained from ATCC (CCL243). T cells were obtained from Stemcell Technologies (Cat# 70500-Full, Lot# 1000079238). GM24631 cells were obtained from Coriell.
Authentication	Cell lines obtained from ATCC and Stemcell Technologies were not internally authenticated
Mycoplasma contamination	Cells were not tested for mycoplasma contamination
Commonly misidentified lines (See ICLAC register)	Used cells are not commonly misidentified

Flow Cytometry

Plots

Confirm that:

☒ The axis labels state the marker and fluorochrome used (e.g. CD4-FITC).

☒ The axis scales are clearly visible. Include numbers along axes only for bottom left plot of group (a 'group' is an analysis of identical markers).

☐ All plots are contour plots with outliers or pseudocolor plots.

☒ A numerical value for number of cells or percentage (with statistics) is provided.

Methodology

Sample preparation	Please refer to materials and methods
Instrument	Attune NXT Acoustic Focussing Cytometer, Model: AFC2 (Thermo Fisher)
Software	FlowJo (v10.4, FlowJo LLC)
Cell population abundance	Approximately 2e5 T cells per well were harvested for flowcytometry
Gating strategy	Gating strategy is shown in Supplementary Figure 10.

☒ Tick this box to confirm that a figure exemplifying the gating strategy is provided in the Supplementary Information.
