# Supplementary material for: Compact zinc finger architecture utilizing toxin-derived cytidine deaminases for highly efficient base editing in human cells
Source: Nat Commun. 2024 Feb 15;15:1181. doi: 10.1038/s41467-024-45100-w (PMC10869815; doi:10.1038/s41467-024-45100-w)
Supplement: Supplementary file 3 — Description of Additional Supplementary Files [file 41467_2024_45100_MOESM3_ESM.pdf]

Title: Supplementary Data 1

Description: DddA-G1333-derived ZF-CBEs targeting CCR5.

Title: Supplementary Data 2

Description: DddA-G1333-derived ZF-CBE-nickases targeting CCR5.

Title: Supplementary Data 3

Description: Split variants of DddA-derived ZF-CBEs targeting CCR5.

Title: Supplementary Data 4

Description: DddA-G1404-derived ZF-CBEs targeting CIITA.

Title: Supplementary Data 5

Description: DddA-G1404-derived ZF-CBE-nickases targeting CIITA.

Title: Supplementary Data 6

Description: TDD-derived ZF-CBEs targeting CIITA: TDD1 to TDD19.

Title: Supplementary Data 7

Description: TDD-derived ZF-CBEs targeting CIITA: TDD21 to TDD31.

Title: Supplementary Data 8

Description: TDD14-derived ZF-CBEs at additional CIITA sites.

Title: Supplementary Data 9

Description: ZF-Deaminase linker study with TDD6 and TDD14-derived ZF-CBEs targeting CIITA.

Title: Supplementary Data 10

Description: ZF-CBE-nickases targeting CIITA in K562 cells: PCR-based NGS assay.

Title: Supplementary Data 11

Description: ZF-CBE-nickases targeting CIITA in T cells: Cell viability assay.

Title: Supplementary Data 12

Description: ZF-CBE-nickases targeting CIITA in T cells: PCR-based NGS assay.

Title: Supplementary Data 13

Description: ZF-CBE-nickases targeting CIITA in T cells: FACS assay.

Title: Supplementary Data 14

Description: Base editing measurements at CIITA for samples used for genome-wide specificity assay.

Title: Supplementary Data 15

Description: pDNA sequences

Title: Supplementary Data 16

Description: TDD annotations

Title: Supplementary Data 17

Description: Construct annotations

Title: Supplementary Data 18

Description: Primers

Title: Supplementary Data 19

Description: Growth media

Title: Supplementary Data 20

Description: rhAmpSeq™ DddA panel amplicon info

Title: Supplementary Data 21

Description: rhAmpSeq™ TDD14 panel amplicon info

Title: Supplementary Data 22

Description: rhAmpSeq™ shared DddA and TDD14 panel amplicon info

Title: Supplementary Data 23

Description: Unedited sample genome-wide specificity assay results

Title: Supplementary Data 24

Description: DddA-G1404 genome-wide specificity assay results

Title: Supplementary Data 25

Description: TDD14-L4/L26 genome-wide specificity assay results

Title: Supplementary Data 26

Description: rhAmpSeq™ assay results

Title: Supplementary Data 27

Description: Performance of ZF-CBE-Nickases at EMILIN2

Title: Supplementary Data 28

Description: Performance of ZF-CBE-Nickases at TRAM1L1

Title: Supplementary Data 29

Description: Performance of ZF-CBE-Nickases at COL5A1

Title: Supplementary Data 30

Description: Performance of ZF-CBE-Nickases at HBB.

Title: Supplementary Data 31

Description: EMILIN2, TRAM1L1, COL5A1, and HBB ZF-CBE performance in T cells: Cell viability assay.

Title: Supplementary Data 32

Description: EMILIN2, TRAM1L1, COL5A1, and HBB ZF-CBE performance in T cells: Base Editing

Title: Supplementary Data 33

Description: Design of EMILIN2, TRAM1L1, COL5A, and HBB ZF-CBE-Nickase constructs, and ZF-free deaminase constructs.

Title: Supplementary Data 34

Description: ZF-free TDD rhAmpSeq™ assay results.
